# Selective Accumulation of Pro-Inflammatory T Cells in the Intestine Contributes to the Resistance to Autoimmune Demyelinating Disease

**DOI:** 10.1371/journal.pone.0087876

**Published:** 2014-02-04

**Authors:** Kerstin Berer, Marina Boziki, Gurumoorthy Krishnamoorthy

**Affiliations:** Department of Neuroimmunology, Max Planck Institute of Neurobiology, Martinsried, Germany; Friedrich-Alexander University Erlangen, Germany

## Abstract

Myelin-specific, pro-inflammatory T_H_17 cells are widely regarded as the drivers of experimental autoimmune encephalomyelitis (EAE), an animal model for Multiple sclerosis (MS). The factors, responsible for the generation and maintenance of T_H_17 cells as well as their participation in the pathogenic cascade leading to the demyelinating disease, have been studied extensively. However, how these harmful autoreactive cells are controlled *in vivo* remains unclear. By comparing TCR transgenic mice on a disease susceptible and a disease resistant genetic background, we show here that pathogenic T_H_17 cells are sequestered within the intestine of spontaneous EAE resistant B10.S mice. Disease resistant B10.S mice harbored higher frequencies of T_H_17 cells in the intestine compared to EAE susceptible SJL/J mice. Moreover, transferred T_H_17 cells selectively migrated to intestinal lymphoid organs of B10.S mice. The sequestration of T_H_17 cells in the gut was partially dependent on the gut homing receptor α4β7-mediated adhesion to the intestine. Administration of α4β7 blocking-antibodies increased the peripheral availability of T_H_17 cells, resulting in increased EAE severity after immunization in B10.S mice. Together, these results support the concept that the intestine is a check-point for controlling pathogenic, organ-specific T cells.

## Introduction

Evidence suggests that autoreactive T cells are commonly present in the healthy immune repertoire, but are kept in check by numerous tolerance mechanisms. Although several details of the tolerance mechanisms have yet to be elucidated, mechanisms including negative selection of autoreactive T cells in the thymus, ignorance, anergy, cytokine immune deviation, tolerogenic antigen presenting cells and induction of regulatory cells have been demonstrated to be involved in mediating self-tolerance [1], [2]. Understanding the mechanisms of self-tolerance, which limits the aberrant activation of self-reactive T cells, is crucial for the development of strategies to treat autoimmune diseases, like Multiple sclerosis (MS).

MS is an extremely complex autoimmune disease caused by various cellular and molecular mechanisms. From the animal models of MS, it became evident that distinct T helper cell subsets, such as IFN-γ-producing T_H_1 and IL-17-producing T_H_17 cells, either alone or in combination are capable of mediating a neurological disease in animals, resembling the human disease [3]–[6]. However, recently, considerable efforts focused on the role of T_H_17 cells in EAE as well as MS, due to their close association with several other autoimmune diseases [7]. Moreover, T_H_17 cells are crucial for controlling the invasion of pathogenic microorganisms and play an important role in intestinal immune homeostasis [8]. In EAE studies, IL-17-deficient mice show attenuated disease symptoms [9], while neutralization of IL-17 during EAE induction greatly delayed the development and reduced the severity of the disease [10]. In addition, transfer of polarized T_H_17 cells induced neurological disease, supporting the idea that this T helper cell subset plays an important role in EAE pathogenesis [3], [6]. Classical T_H_17 cells secrete IL-17a, IL-17f, IL-21 and IL-22 as their key effector cytokines and make use of the chemokine receptor CCR6 to enter the target tissues [11]–[13]. *In vitro* as well as *in vivo* generation of T_H_17 cells requires the induction of their master transcription factor retinoic acid-related orphan receptor-γt (ROR-γt) [14]. Despite the extensive knowledge about the generation and maintenance of T_H_17 cells, how these cells are regulated *in vivo* during autoimmune disease settings needs further investigation.

In this report, we compared the development of spontaneous EAE in myelin-specific TCR transgenic mice on the disease-susceptible SJL/J genetic background with animals on the disease-resistant B10.S background. We found that the pro-inflammatory, myelin-reactive T_H_17 cells were enriched in the intestine of B10.S mice, but failed to reach the peripheral immune organs, resulting in the absence of spontaneous EAE in B10.S mice. The release of intestine-sequestered T_H_17 cells by treating B10.S mice with α4β7-specific monoclonal antibodies worsened EAE. We propose that the immune tolerance against myelin-specific self-antigens could be achieved by selective sequestration of pro-inflammatory T cells in the intestine.

## Results

### Absence of spontaneous EAE in MOG-specific TCR transgenic B10.S mice

**Figure 1:**
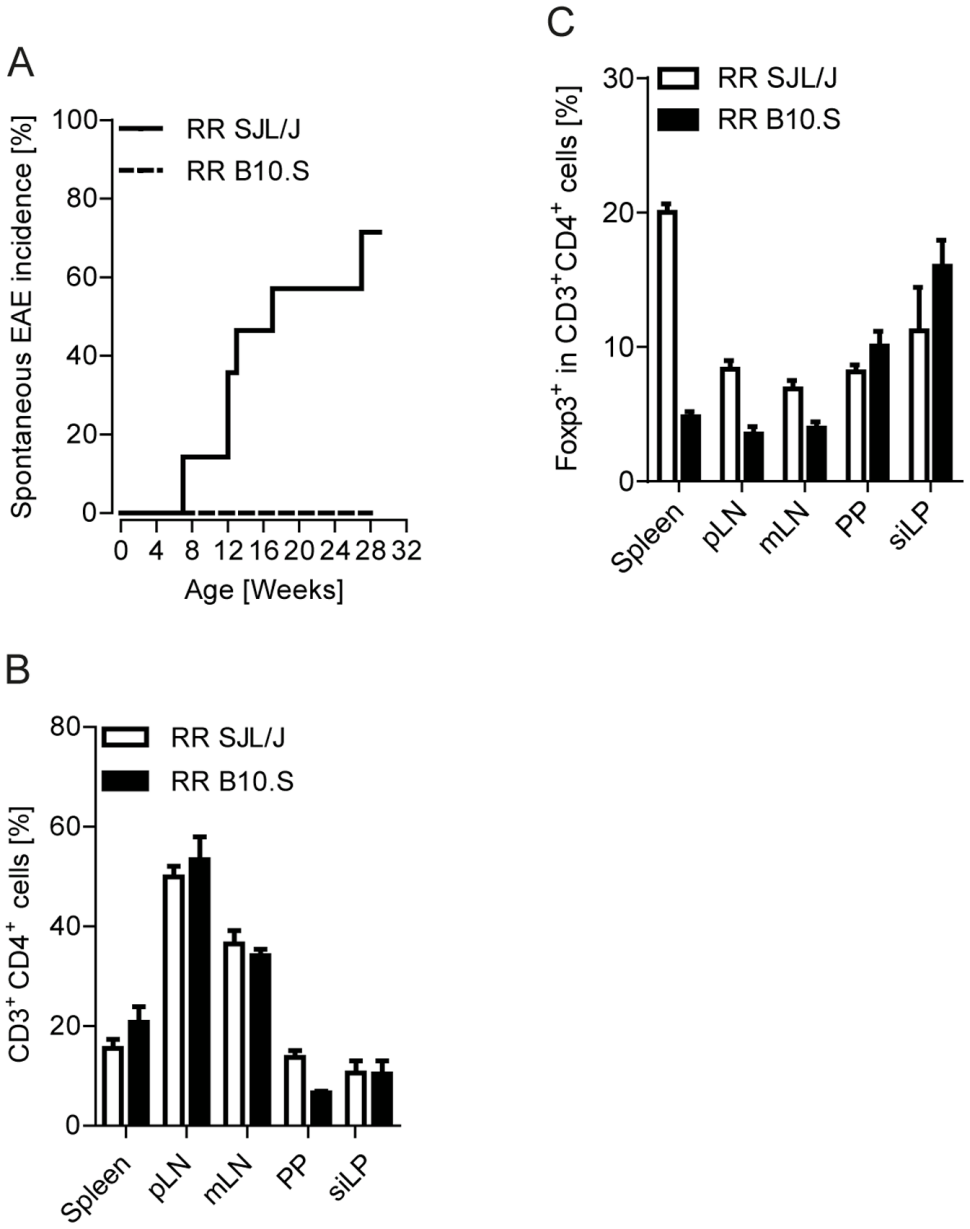
Absence of spontaneous EAE in RR B10.S mice. A. Incidence of spontaneous EAE in a cohort of RR SJL/J (n = 14) or RR B10.S mice (n = 17). B. Frequencies of CD3^+^ CD4^+^ T cells in spleen, pooled axillary and inguinal lymph nodes (pLN), mesenteric lymph nodes (mLN), Peyer's patches (PP) and small intestinal lamina propria (siLP) were measured by flow cytometry. C. Frequencies of Foxp3^+^ CD3^+^ CD4^+^ regulatory T cells in the indicated organs were measured by flow cytomtery. Results are from n  = 4–6 mice per group. Data were pooled from 2–3 different experiments (B–C). Error bars indicate SEM (B–C).

We recently described a transgenic mouse strain that expresses a myelin oligodendrocyte glycoprotein (MOG)-specific T cell receptor (TCR) on the SJL/J genetic background (RR mice). The TCR was derived from an encephalitogenic T cell clone generated from recombinant MOG protein (rMOG) immunized wild-type SJL/J mice [15]. To get insights into the mechanisms of precipitation of spontaneous autoimmunity and tolerance, we backcrossed RR SJL/J animals to MHC congenic B10.S mice. Similar to SJL/J mice, B10.S mice harbor the MHC class II allele I-A^s^, but on the C57BL/10 background. Although RR SJL/J and RR B10.S mice express the same pair of TCR Vα8.3 and Vβ4 chains on their CD4^+^ T cells, the incidence of spontaneous EAE was strikingly different. While more than 80% of the RR SJL/J cohort presented spontaneous EAE symptoms between 6–28 weeks of age, none of the B10.S mice displayed any clinical signs of disease (**Figure 1A**). In our colony of more than 300 RR B10.S mice maintained during a period of 5 years, we noted only 3 mice with spontaneous EAE symptoms, accounting for less than 1% of disease incidence.

### T cell and APC functions in B10.S mice

To determine whether resistance to spontaneous EAE in B10.S animals is due to alterations in T cells, we analyzed T cell populations in the peripheral lymphoid organs. Flow cytometric analysis of the peripheral lymphoid organs showed that the frequencies of CD4^+^ T cells in various lymphoid organs of B10.S mice were comparable to those of their SJL/J counterparts (**Figure 1B**). The resistance to EAE in B10.S has been attributed to an increase in the frequencies of Foxp3^+^ regulatory T cells (Treg cells) [16]. However, the frequencies of Treg cells were comparable in most of the lymphoid organs of RR SJL/J and B10.S mice. In the spleen of RR B10.S mice, we even observed 4-fold lower frequencies of Treg cells than in SJL/J animals (**Figure 1C**). Thus, we conclude that Treg cells do not vitally contribute to the observed EAE resistance in RR B10.S mice.

**Figure 2:**
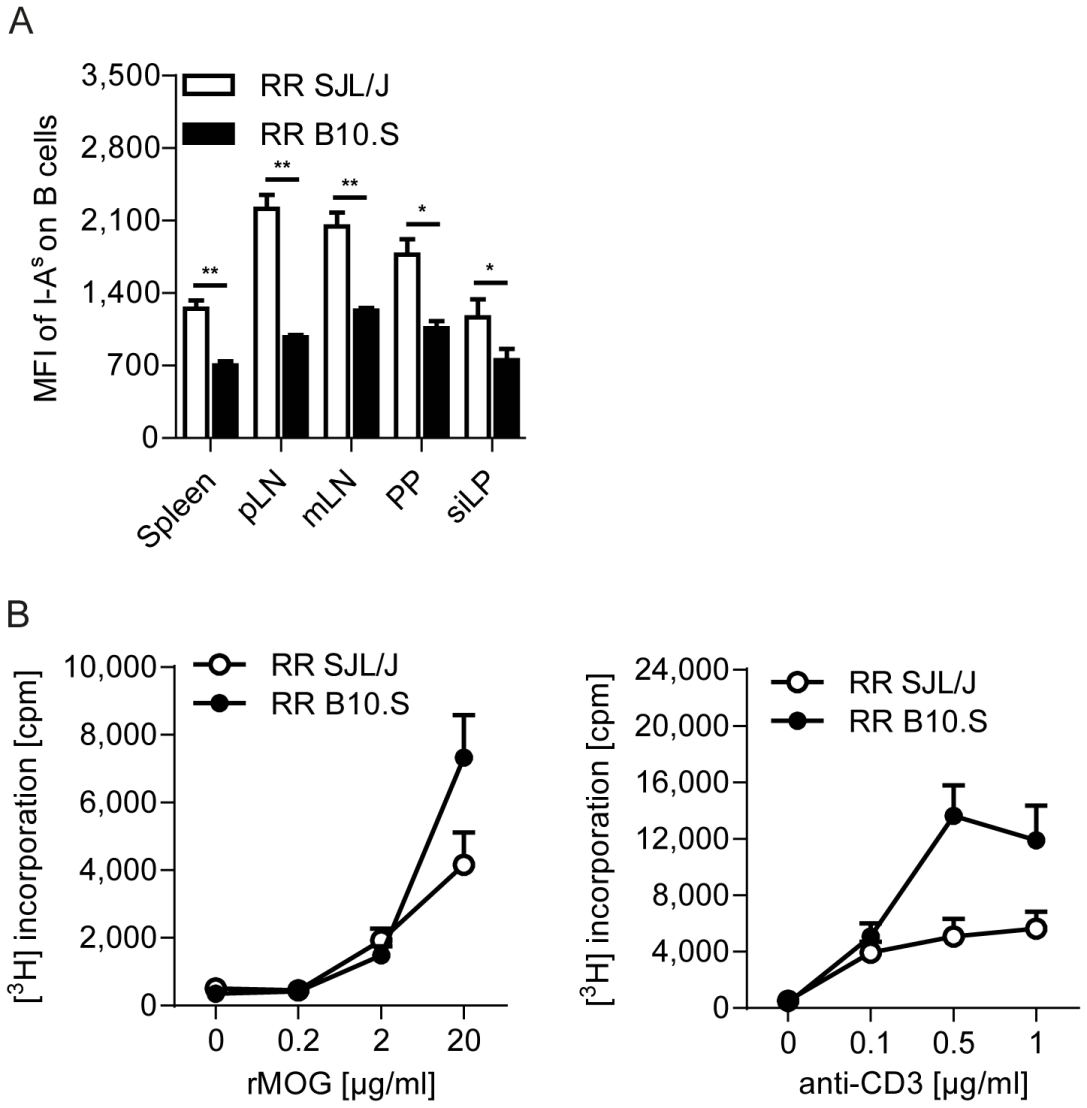
Activation of T cells is not affected by lower MHC class II expression on APCs of B10.S mice. A. Expression of the MHC class II molecule I-A^s^ on B cells was determined by flow cytometry in various lymphoid organs of RR SJL/J and B10.S mice. Bar graphs show the mean fluorescent intensity (MFI) + SEM of I-A^s^ on the gated B220^+^ population. *, p<0.05; **, p<0.01 (Mann-Whitney U test). Results are from n = 5–7 mice per group from 3 different experiments. B. Splenocytes from RR SJL/J or RR B10.S mice were isolated and their proliferative response to the indicated concentrations of rMOG or anti-CD3 tested *in vitro*. Data are shown as mean counts per minute (cpm) with error bars indicating SEM. Data were pooled from 3 different experiments.

Next, we examined the functional status of the antigen presenting cells (APCs) in B10.S mice. A previous report showed that APCs from B10.S mice express lower amounts of MHC class II [17]. Flow cytometric analysis of MHC class II (I-A^s^) on B cells from various lymphoid organs confirmed the lower MHC class II expression levels on B10.S APCs compared to APCs from SJL/J mice (**Figure 2A**). In addition, we evaluated the expression levels of several surface markers related to co-stimulation, such as CD86, PDL-1 or ICOS-L. However, there were no significant differences in the expression of these co-stimulatory/co-inhibitory molecules on splenic B cells (**Figure S1**). Stimulation of the APCs innate immune response by microbial products can result in the upregulation of MHC class II on the surface of B10.S APCs [17]. However, *in vivo* activation of APCs as well as T cells via immunization with rMOG in CFA did not result in a massive upregulation of the MHC class II-expression on APCs from B10.S mice (**Figure S1**). Moreover, in the gut associated lymphoid tissues (GALT), where APCs constantly encounter microbial products due to their close proximity to the commensal microbiota, B10.S B cells expressed lower amounts of MHC class II (**Figure 2A**).

We subsequently analyzed if the lower MHC class II-expression on APCs from B10.S mice would result in defective T cell activation and thus, provide an explanation for the absence of spontaneous EAE in RR B10.S mice. However, despite their lower MHC class II-expression, B10.S APCs were capable of processing and presenting the cognate antigen MOG as efficiently to T cells as APCs from SJL/J mice. Moreover, T cells from B10.S mice showed a higher proliferative response to anti-CD3 stimulation than SJL/J T cells, suggesting no intrinsic or extrinsic defect in T cell activation or functional capacity of APCs in B10.S mice (**Figure 2B**). Taken together, we conclude that the resistance to spontaneous EAE is not due to aberrant functional alterations of neither T cells nor APCs in RR B10.S mice.

### Impaired accumulation of T_H_17 cells in the peripheral lymphoid organs of B10.S mice

**Figure 3:**
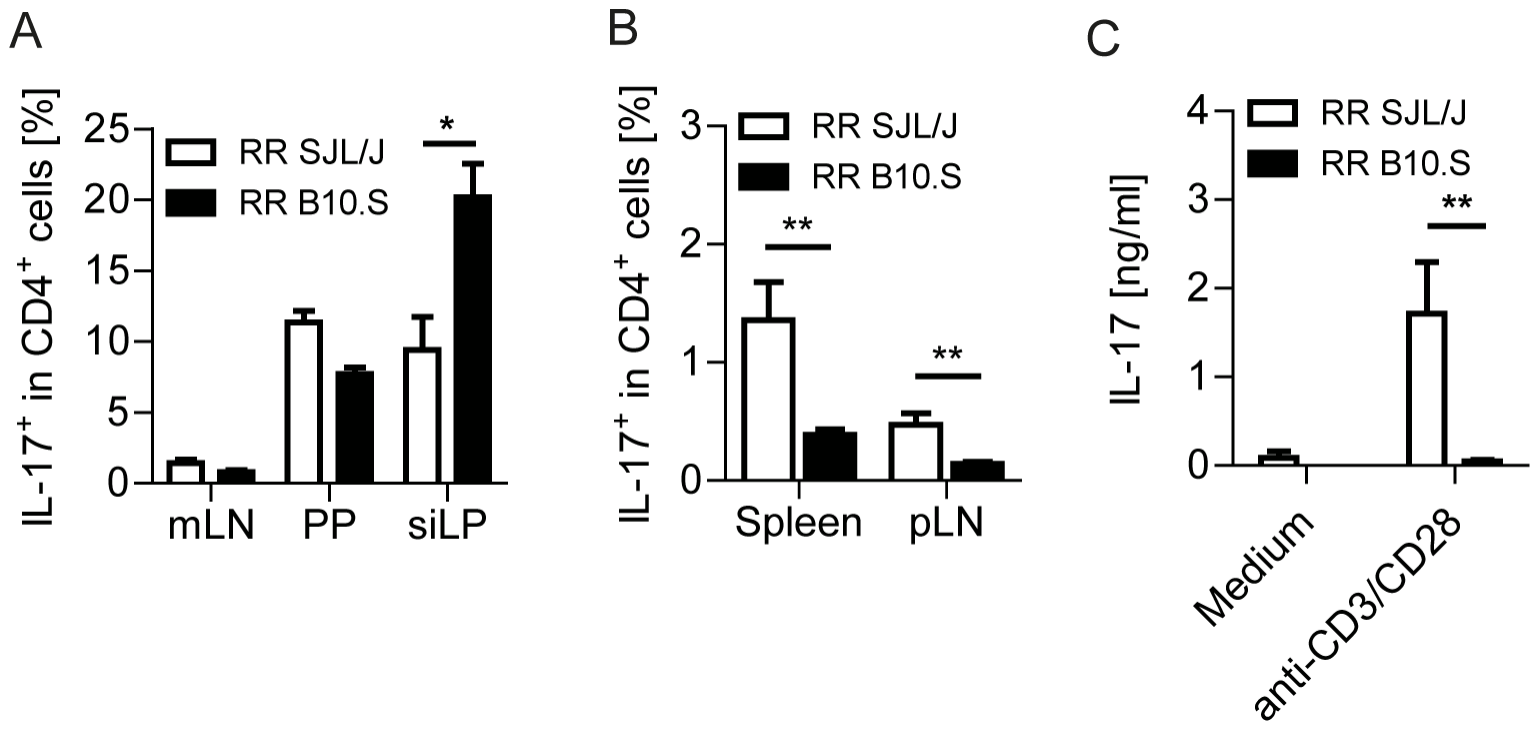
B10.S T_H_17 cells fail to accumulate in peripheral lymphoid organs. A. Flow cytometric analysis of IL-17 expression by CD4^+^ T cells in the gut associated lymphoid tissues. Data represent the percentage of cytokine producing cells in the gated CD4^+^ population. *, p<0.05; (Mann-Whitney U test). B. Flow cytometric analysis of IL-17 expression by T cells in the peripheral lymphoid organs. Data represent the percentage of cytokine producing cells in the gated CD4^+^ population. **, p<0.01 (Mann-Whitney U test). Results are from n = 5–6 mice per group. Data were pooled from 2–3 different experiments (A–B). Error bars indicate SEM (A–B). C. Cytokine production by splenocytes from RR SJL/J or B10.S mice. After a 72 hours culture period, production of IL-17 by RR SJL/J or B10.S spleen cells in response to medium alone or anti-CD3/anti-CD28 stimulation were measured by ELISA. Bars depict mean + SEM. **, p<0.01 (Mann-Whitney U test).

Having excluded any general defect in T cell functionality, we sought to characterize the distribution of T helper cell-subsets in B10.S mice. EAE induction in mice has been attributed to distinct T helper cell subsets, which produce either IFN-γ or IL-17, T_H_1 or T_H_17 cells, respectively [3]. Recent set of seminal papers showed that at steady state, distinct components of the gut commensal microbiota are necessary to trigger T_H_17 cell differentiation, which subsequently contribute to the development of autoimmune diseases [18]–[20]. Moreover, we were previously able to demonstrate that spontaneous EAE development in RR SJL/J mice is critically dependent on an intact commensal microbiota, which activates and directs CD4^+^ T cells to differentiate into pathogenic T_H_17 cells [21]. To validate the T_H_17-inducing potential of the gut microbiota of B10.S mice, we compared the frequency of this T helper cell subset in the GALT of RR SJL/J and B10.S mice using flow cytometric analysis. The frequencies of IL-17-producing CD4^+^ T cells were elevated in the small intestinal lamina propria (LP) of B10.S mice compared to their SJL/J counterparts (**Figure 3A**). The increased frequency of LP T_H_17 cells, however, was not due to a higher abundance of T_H_17 cell-inducing segmented filamentous bacteria (SFB) in the intestines of B10.S mice, since colonization levels of SFB were comparable between SJL/J and B10.S animals (**Figure S2**).

Next, we checked the fate of the pro-inflammatory T_H_17 cells, which are triggered in the intestine. Interestingly, in SJL/J mice, T_H_17 cells, which were activated in the gut, populate peripheral lymphoid organs and form a pool of pathogenic CD4^+^ T cells, ready to invade the central nervous system and mediate neurological disease [21]. Direct *ex vivo* analysis of T_H_17 cells in B10.S mice, however, revealed that despite the higher abundance of T_H_17 cells in the GALT, frequencies of T_H_17 cells were greatly reduced in spleen and lymph nodes (**Figure 3B**). The lower IL-17 production by splenic T cells from B10.S mice was confirmed by measuring the recall response after *in vitro* stimulation with anti-CD3/CD28 antibodies (**Figure 3C**).

### Mechanisms of accumulation of T_H_17 cells in the intestine and EAE development

**Figure 4:**
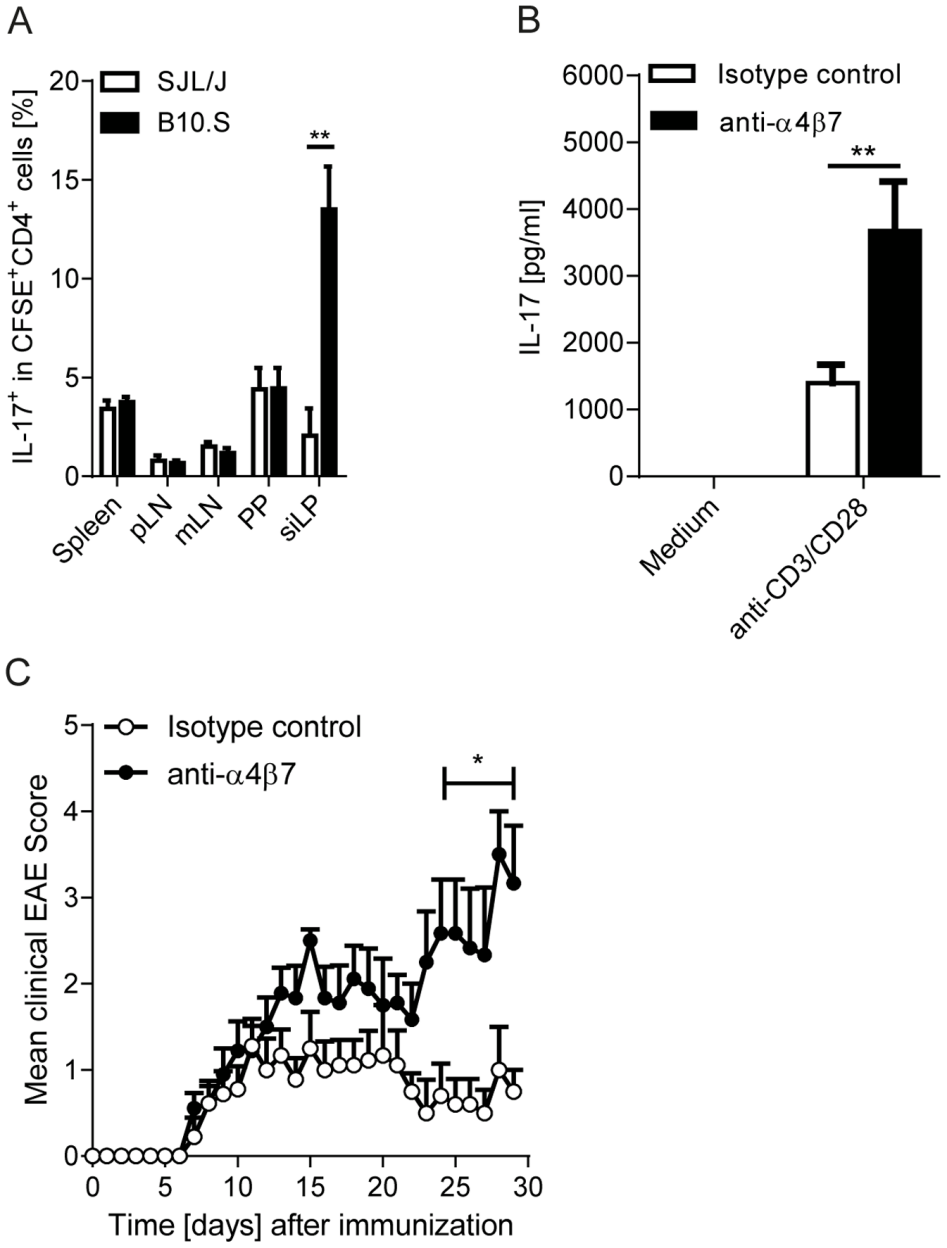
Integrin α4β7 mediated accumulation of T_H_17 cells in the intestine of RR B10.S mice. A. Enhanced migration of T_H_17 cells to the intestine. Flow cytometric analysis of transferred CFSE-labeled T_H_17 cells in various lymphoid organs. Data represent the percentage of IL-17^+^ T cells in the gated CFSE^+^ CD4^+^ population. **, p<0.01 (Mann-Whitney U test). Results are from n = 5–6 mice per group. Data were pooled from 2–3 different experiments. B. Blockade of α4β7 increases peripheral availability of IL-17-producing T cells. RR B10.S mice were treated weekly twice with anti-α4β7 or isotype control antibodies for two consecutive weeks. Splenocytes were stimulated *in vitro* with anti-CD3/anti-CD28 antibodies. After a 72 hours culture period, production of IL-17 was measured by ELISA. Bars show mean + SEM. **, p<0.01 (Mann-Whitney U test). n = 11–12 mice per group. Data were from 3 independent experiments. C. Effect of blockade of α4β7 on EAE pathogenesis. RR B10.S mice were immunized with rMOG in CFA and treated weekly twice with anti-α4β7 or isotype control antibodies. Mean clinical scores are depicted. *, p<0.05. n = 9–11 mice per group. Data were from 3 independent experiments.

We next wanted to identify the mechanism(s) responsible for the accumulation of T_H_17 cells in the intestine and hence, for the lack of a peripheral T_H_17 cell-pool in B10.S mice. The specific enrichment might be the result of either impaired emigration of T_H_17 cells from the intestine after their induction by gut commensals or the increased migration from the periphery to the intestine [22]. We transferred *in vitro* polarized CFSE-labeled T_H_17 cells into naïve SJL/J or B10.S recipients and traced their homing to various lymphoid organs. Whereas we detected comparable frequencies of T_H_17 cells migrating to peripheral lymphoid organs, the intestine, i.e. the lamina propria, of B10.S mice accumulated higher frequencies of transferred T_H_17 cells than the intestine of SJL/J recipients (**Figure 4A**).

Gut-tropic T cells express the integrin receptor α4β7, whose ligand MAdCAM-1 is expressed on post capillary venules in the intestinal LP [23]. To test the role of the gut homing receptor α4β7 in intestinal accumulation of T_H_17 cells, we treated RR B10.S mice with a blocking antibody to α4β7. Interestingly, blockade of α4β7 led to an enhanced production of IL-17 in the spleen (**Figure 4B**), suggesting that the α4β7 integrin receptor at least partially contributes to the migration and retention of T_H_17 cells in the B10.S intestine. In addition, enhanced peripheral availability of T_H_17 cells in B10.S mice through blockade of α4β7 resulted in an increased EAE severity (**Figure 4C**). In summary, these data suggests that while pathogenic T_H_17 cells are efficiently induced in the intestine of B10.S mice, they fail to accumulate in peripheral lymphoid organs, a process partly dependent on the α4β7 integrin-mediated adhesion to the intestine.

## Discussion

In this study, we describe a mechanism of tolerance to the development of spontaneous EAE. We compared transgenic mice that bear the same rearranged MOG-specific TCR in the EAE-susceptible SJL/J or the EAE-resistant B10.S genetic background. A lack of spontaneous EAE in B10.S mice in contrast to SJL/J animals led us to investigate the cellular mechanisms responsible for the resistance to the disease. We found that the pathogenic T_H_17 cells were contained within the intestine of B10.S mice. The retention of T_H_17 cells is presumably mediated by the enhanced migration as well as interaction of their gut homing integrin α4β7 with MAdCAM-1, expressed in the intestine.

The genetic background profoundly influences the purging of the autoimmune T cell repertoire. It is assumed that the presentation of low avidity self-peptide – MHC complexes in the thymus promotes the development of potentially autoreactive T cells, which may escape thymic negative selection. This is reflected by the fact that various inbred strains differ in their response to distinct myelin antigens, thus presenting varied immune and clinical responses to these autoantigens [24]. To avoid potential artifacts due to the expansion of a different T cell repertoire, we used transgenic mice expressing a MOG-specific T cell receptor, which recognizes the MOG-peptide 92–106 in the context of I-A^s^, but on two different genetic backgrounds.

RR SJL/J mice develop high incidence of spontaneous EAE, whereas B10.S mice remained free of clinical disease symptoms. EAE resistance offers a unique opportunity to study the regulatory mechanisms that control autoimmunity. Initially, we speculated that MOG-specific T cells might be different in these two mouse strains, but flow cytometric analysis of various peripheral and intestinal lymphoid organs revealed no abnormalities in T cell development in B10.S mice with frequencies of CD4^+^ CD3^+^ cells being comparable between the two mouse strains. APCs play an important role in determining the type and degree of T cell responses by providing co-stimulatory signals and establishing the local cytokine-milieu. We confirmed an earlier report describing lower basal levels of MHC class II molecules on APCs of B10.S mice [17]. Nevertheless, B10.S APCs were still efficient processors and presenters of MOG protein to T cells.

Various reasons were suggested, explaining the resistance of B10.S mice to EAE-development after immunization with MBP or PLP peptides. Differences in the blood-brain barrier permeability between B10.S and SJL/J mice were demonstrated, with later reports unable to confirm these findings [25], [26]. Increased frequencies of regulatory T (Treg) cells have been observed in naïve B10.S mice [27]. Depletion of Treg cells partly restored EAE susceptibility with a concomitant increase in the production of IFN-γ, IL-6 and IL-17 [16]. We, however, did not observe any changes in the Treg cells in our TCR transgenic mice on B10.S background. This may be due to the fact that this particular TCR may not favor Treg cell differentiation on certain genetic backgrounds. Previous reports also indicated a defect in T helper cell responses in B10.S mice [28]. The resistance to EAE in B10.S mice was partially associated with the reduced production of the pro-inflammatory cytokine IFN-γ or the elevated production of IL-4 and IL-10 after immunization [29], [30].

While studying immunization models, the effect of the adjuvants on skewing the T helper cell responses cannot be ruled out, we therefore decided to investigate the phenotypes of the T helper cell subsets in a “natural” (unimmunized) state. Striking differences were observed in the frequencies of IL-17-producing T_H_17 cells. B10.S mice contained far lower frequencies of T_H_17 cells in all secondary peripheral lymphoid organs compared to their SJL/J counterparts. To confirm that these cells actually produce low levels of their specific pro-inflammatory cytokine, IL-17, we measured the *in vitro* recall response. B10.S mice selectively lacked the production of IL-17. At steady state, T_H_17 cells are induced in the intestine, which is essential for maintaining mucosal immune homeostasis [14]. The differentiation of T_H_17 cells in the mucosal tissue is controlled at least in parts by specific commensal gut microbial species, segmented filamentous bacteria (SFB), respectively [18], [31], [32]. Moreover, commensal microbiota induced T_H_17 cells have been shown to be critically regulating the susceptibility to autoimmune diseases [19], [33]. Recently, we showed that spontaneous EAE development in the susceptible SJL/J background requires the activation and differentiation of T cells into pro-inflammatory lineages in the gut [21]. While analyzing the GALT, we found that in contrast to the peripheral lymphoid tissues, B10.S mice harbored higher frequencies of T_H_17 cells in the small intestinal lamina propria than SJL/J animals. However, the T_H_17 cell-inducing load of the segmented filamentous bacteria was similar between SJL/J and B10.S mice. This finding suggests that B10.S mice are indeed capable of mounting a robust T_H_17 response, but these T cells fail to reach the peripheral lymphoid organs. To investigate whether T_H_17 cells may have an enhanced migratory affinity towards the intestine of B10.S mice, we transferred T_H_17 cells into naïve SJL/J and B10.S recipients. Interestingly, we found elevated numbers of T_H_17 cells in the intestine of B10.S mice compared to SJL/J recipients, suggesting that the B10.S gut harbors a milieu, which is attractive for T_H_17 cells.

It is well appreciated that chemokines and their receptors along with adhesion molecules control the homing pattern of lymphocytes to lymphoid and non-lymphoid tissues. Gut tropism of T cells are mediated by the integrin receptor α4β7 and the chemokine receptor CCR9 that is induced by the gut associated CD103^+^ dendritic cells [23], [34]. Our experiments showed that the blocking of the gut homing integrin receptor α4β7-mediated adhesion to the intestine with a monoclonal antibody increased the peripheral availability of T_H_17 cells, resulting in a more severe EAE. Our results suggest that multiple mechanisms are employed to target effector pathogenic T_H_17 cells to the intestinal mucosa, making them unavailable to participate in pro-inflammatory responses at peripheral sites.

In summary, we show here that the resistance to spontaneous EAE in B10.S mice is partly due to enhanced accumulation of activated MOG-specific T_H_17 cells in the intestine leading to a reduced frequency of this T helper cell subset in the peripheral repertoire. Understanding the mechanisms that promote the accumulation of pro-inflammatory T_H_17 cells may help in devising new therapeutic approaches to control MS.

## Materials and Methods

### Animals

A wild-type B10.S mice colony was established from a breeding pair obtained from the McLaughlin Research Institute (Montana, USA). TCR transgenic RR SJL/J mice [15] were backcrossed for more than 9 generations to B10.S mice. Mice were bred at the animal facility of the Max Planck Institute of Neurobiology (Martinsried, Germany). All animal procedures were approved by the Regierung von Oberbayern (Munich, Germany).

### Immunization and evaluation of EAE

Mice were immunized subcutaneously with 200 µg rMOG emulsified in Freund's adjuvant supplemented with 5 mg/ml Mycobacterium tuberculosis (strain H37Ra; Difco) (CFA). 200 ng of pertussis toxin (List Biological Laboratories) were injected intraperitoneally on the day of immunization and 48 hours later. In addition, mice were injected with 250 µg of anti-α4β7 (DATK 32) or isotype control antibodies weekly twice. Clinical signs of EAE were assessed daily according to the standard 5 point scale [15].

### Proliferation assay

Splenocytes from RR SJL/J or RR B10.S mice were cultured in the presence of the indicated concentration of rMOG or anti-CD3/anti-CD28 antibodies (BD Pharmingen). The proliferative response was measured by the incorporation of [^3^H]thymidine during the final 16 hours of a 72 hour culture period. Results are expressed as mean thymidine uptake (cpm) of triplicate cultures.

### CFSE-labeled T_H_17 cell transfer

Polarization of splenic CD4^+^ T cells towards T_H_17 cells was performed as previously described [3]. After 6 days in culture, T_H_17 cells were purified by Nycoprep (Axis-Shield). Purified T cells were labelled for 10 min at 37°C with 5 mM CFSE (Life Technologies) in PBS containing 1% fetal bovine serum (FBS). Subsequently, cells were washed twice in ice-cold PBS. 5×10^6^ CFSE-labelled T_H_17 cells were injected intravenously into wild-type SJL/J or B10.S mice. The frequencies of CFSE^+^ T_H_17 cells were measured by flow cytometry after 3 days.

### Cell isolation and flow cytometry

Single-cell suspensions were prepared from spleen, pooled peripheral lymph nodes (axillary plus inguinal) or Peyer's patches by mechanical disruption via forcing through 40 µm cell strainers (BD Biosciences). Lamina propria lymphocytes were isolated as previously described [21]. For detection of cell surface markers, cells were stained in FACS buffer (PBS containing 1% BSA and 0.1% NaN_3_) with the following fluorochrome-labeled monoclonal antibodies: anti-CD4 (RM4-5), anti-CD3 (145-2C11), anti-B220 (RA3-6B2), anti-I-A^q^ (KH116), anti-CD86 (GL1), anti-PDL-1 (MIH5), anti-ICOS-L (HK5.3). For intracellular cytokine staining, cells were activated with 50 ng/ml PMA (Sigma) and 500 ng/ml ionomycin (Sigma) in the presence of 5 mg/ml brefeldin A (Sigma) for 4 hours at 37°C. After surface staining, cells were fixed and permeabilized using the Transcription Factor Staining Buffer Set (eBioscience) and stained intracellularly with the following antibodies: anti-IL17 (TC11-18H10) or anti-FoxP3 (FJK-16s). All antibodies were purchased from BD Pharmingen or eBioscience. Cells were acquired on a FACSCalibur or FACSVerse (BD Biosciences) and analysis was performed using FlowJo (TreeStar) software.

### ELISA

Cytokine levels in cell culture supernatants were determined using matching antibody pairs for IL-17 (eBioscience) according to manufacturer's instructions.

### Fecal Bacterial DNA Extraction and qPCR

Bacterial genomic DNA was extracted from fecal pellets using QIAamp DNA Stool mini kit (Qiagen). Real-time PCR targeted to 16S rDNA was performed using Absolute QPCR SYBR Green Mix (Thermo Fisher Scientific) and a 7900HT Real-time PCR System (Applied Biosystems). Values were normalized to total bacteria. The following primer sets were used [35]: total bacteria, ACTCCTACGGGAGGCAGCAGT and ATTACCGCGGCTGCTGGC; *Segmented filamentous bacteria*, GACGCTGAGGCATGAGAGCAT and GACGGCACGGATTGTTATTCA.

## Supporting Information

Figure S1 **Stimulation of the innate immune response does not result in enhanced MHC class II-expression on APCs of B10.S mice.** Expression-levels of MHC class II (I-As) and the co-stimulatory/co-inhibitory molecules (CD86, PDL-1 and ICOS-L) on B cells were determined by flow cytometry in the spleen of naïve wild type SJL/J and B10.S mice and wild-type animals 10 days after immunization with rMOG/CFA. Bar graphs show the mean fluorescent intensity (MFI) + SEM on the gated B220^+^ population. *, p<0.05 (Mann-Whitney U test). Results are from n  = 4–5 mice per group. Data were pooled from 2 independent experiments.
(TIF)

Figure S2 **Fecal SFB content in B10.S and SJL/J mice.** 16S rDNA PCR for the presence of SFB in the feces of RR SJL/J or B10.S mice. Values are shown as relative amount to total bacterial 16S rDNA.
(TIF)
