# Supplementary figures and images for: Selective Accumulation of Pro-Inflammatory T Cells in the Intestine Contributes to the Resistance to Autoimmune Demyelinating Disease
Source: PLoS One. 2014 Feb 4;9(2):e87876. doi: 10.1371/journal.pone.0087876 (PMC3913661; doi:10.1371/journal.pone.0087876)

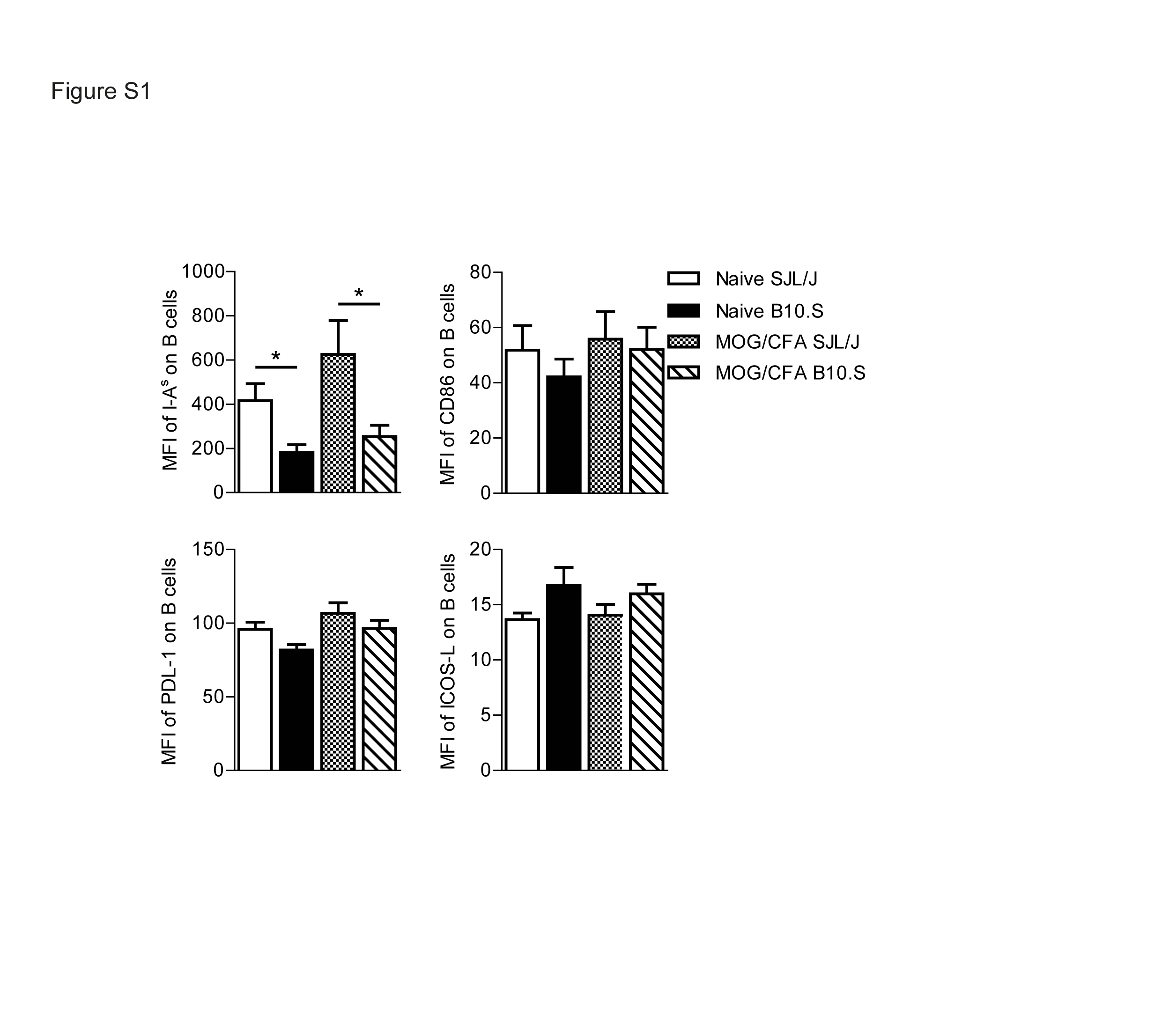

Supplement: Figure S1 — Stimulation of the innate immune response does not result in enhanced MHC class II-expression on APCs of B10.S mice. Expression-levels of MHC class II (I-As) and the co-stimulatory/co-inhibitory molecules (CD86, PDL-1 and ICOS-L) on B cells were determined by flow cytometry in the spleen of naïve wild type SJL/J and B10.S mice and wild-type animals 10 days after immunization with rMOG/CFA. Bar graphs show the mean fluorescent intensity (MFI) + SEM on the gated B220+ population. *, p<0.05 (Mann-Whitney U test). Results are from n = 4–5 mice per group. Data were pooled from 2 independent experiments. (TIF) [file pone.0087876.s001.tif]

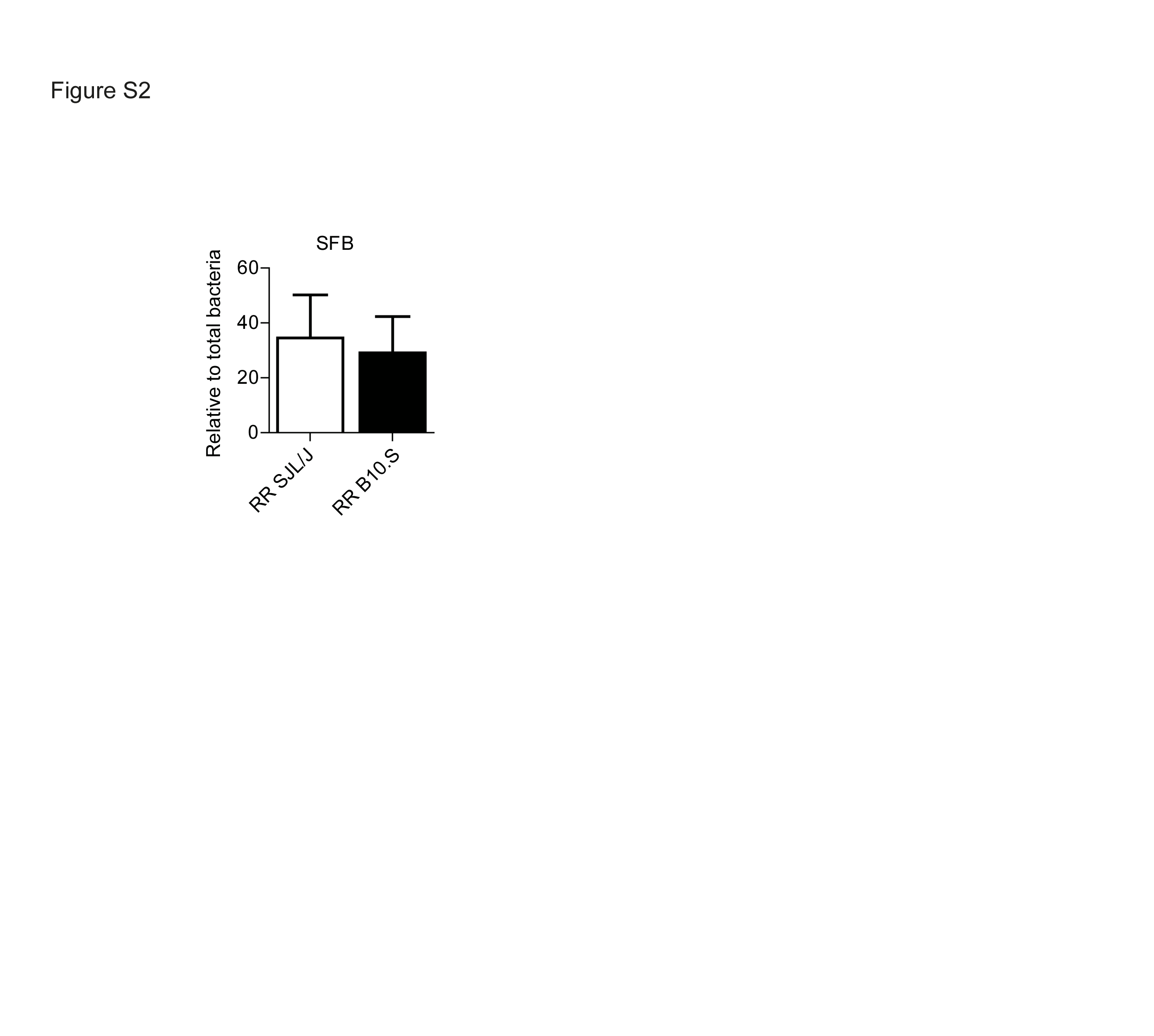

Supplement: Figure S2 — Fecal SFB content in B10.S and SJL/J mice. 16S rDNA PCR for the presence of SFB in the feces of RR SJL/J or B10.S mice. Values are shown as relative amount to total bacterial 16S rDNA. (TIF) [file pone.0087876.s002.tif]
